# The Burden of Diarrheal Diseases and Its Associated Factors among Under-Five Children in Welkite Town: A Community Based Cross-Sectional Study

**DOI:** 10.3389/ijph.2022.1604960

**Published:** 2022-10-12

**Authors:** Deneke Wolde, Genet Asfaw Tilahun, Kehabtimer Shiferaw Kotiso, Girmay Medhin, Tadesse Eguale

**Affiliations:** ^1^ Department of Medical Laboratory Science, College of Medicine and Health Sciences, Wachemo University, Hosssana, Ethiopia; ^2^ Aklilu Lemma Institute of Pathobiology, Addis Ababa University, Addis Ababa, Ethiopia; ^3^ Department of Midwifery, College of Medicine and Health Sciences, Wolkite University, Wolkite, Ethiopia; ^4^ Department of Public Health, College of Medicine and Health Sciences, Worabe University, Worabe, Ethiopia

**Keywords:** diarrhea, prevalence, caregivers, risk factors, Ethiopia

## Abstract

**Objective:** This study assessed the magnitude of diarrhea and associated risk factors among under-five children in Welkite town.

**Methods:** We used a community-based cross-sectional study design. Data collection period was February to March 2021 and 426 parents/guardians of under-five children were the study participants. A structured questionnaire and observation checklist were used to collect the data.

**Results:** The 2 weeks prevalence of diarrhea among under-five years old children was 20.7% (88/426); 95% CI (17.1, 24.6). The child’s mother/caregiver being merchant (AOR: 5.34; 95% CI: 2.1, 13.8) compared to housewife, partial immunization status (AOR: 2.67; 95% CI: 1.2, 5.8), disposing child’s stool into the garbage (AOR: 5.05; 95% CI: 1.1, 23.3) compared to putting in a toilet, not covering water storage materials (AOR: 2.4; 95% CI: 1.2, 4.7) and presence of flies in food preparation area (AOR: 2.24; 95% CI: 1.05, 4.8) were associated with increased odds of having diarrhea.

**Conclusion:** The prevalence of diarrhea among under-five old children is high and it is associated with the occupation of the mothers/caregivers, the immunization status of children, unhygienic water storage condition and non-hygienic household practice.

## Introduction

Diarrheal disease is a major public health problem throughout the world and is responsible for high morbidity and mortality and is among the leading causes of outpatient visits, hospitalization, and the global year of life lost (YLL) in people of all ages [1]. Approximately 1.6 million deaths occur each year globally due to diarrhea with the highest-burden occurring in developing countries and economically disadvantaged regions [2].

Globally, diarrhea contributed to 15% of all under-five deaths [3, 4]. Of all child deaths from diarrhea, 78% occur in the African and Southeast Asian regions [5–7]. In these regions, diarrhea accounts for one in eight deaths among children younger than 5 years per annum [8]. Although the mortality from diarrhea has declined considerably over the past 25 years globally, diarrhea-associated morbidity in sub-Saharan Africa remains unacceptably high [9]. By 2030, it is estimated that 4.4 million children under the age of five will die from infectious diseases annually and that 60% of those deaths will occur in sub-Saharan Africa unless the appropriate measure is taken [10].

Ethiopia is the second-most populous country in Africa with a population of over 110 million and of these; more than 14% are children under-five years of age [11]. Regardless of the interventions undertaken, the burden of diarrheal disease is high, and there is a considerable variation in the prevalence and determinant factors of diarrhea in different localities of the country. Compared to other sub-Saharan African countries, Ethiopia accounts higher incidence of diarrheal diseases contributing to avoidable deaths [12, 13] and the diarrheal disease accounts for 9% of child mortality [14].

Diarrhea has been associated with reduced growth, impaired cognitive function, reduced vaccine efficacy, disruption of physical and educational development in children [15–18]. Factors determining the occurrence of diarrhea are complex, and the relative contribution of each factor varies as a function of the interaction between socioeconomic, environmental, and behavioral variables [15, 19]. The occurrence of diarrhea is associated with unsafe drinking water and poor sanitation in about 90% of the cases [20]. Sub-Saharan Africa showed slower progress in sanitation coverage, reaching 31% in 2015 from 24% in 1990 [21].

Accurate information on prevalence and factors associated with childhood diarrhea in the Welkite town remains virtually unknown. The aim of this study was assess the prevalence of diarrhea in the past 2 weeks and determine the contribution of socioeconomic, environmental, and behavioral factors to the occurrence of diarrhea among under-five children in Welkite town.

## Methods

### Study Area and Setting

**FIGURE 1:**
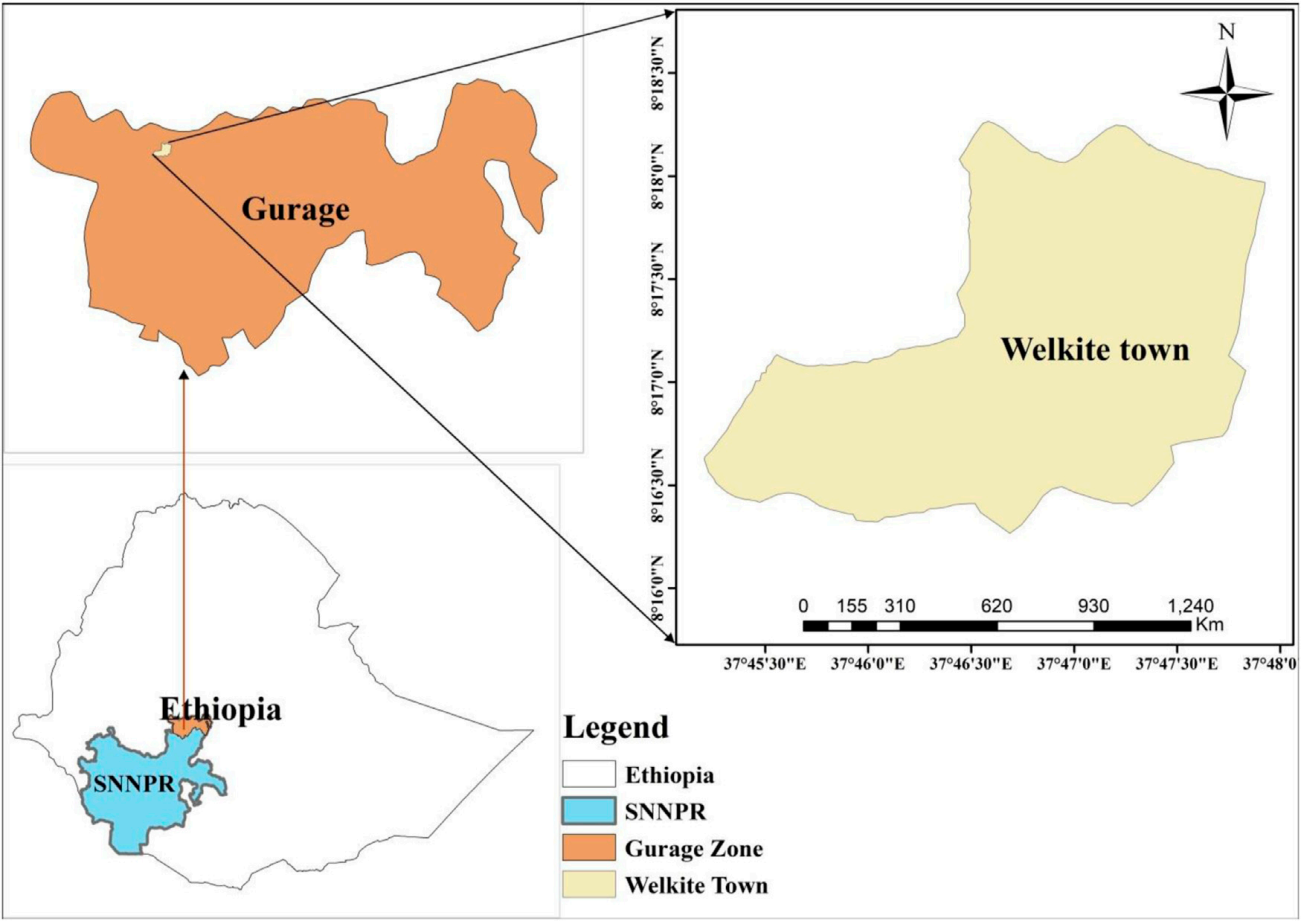
Map of Welkite town, Gurage zone, southern Ethiopia, 2021.

The study was conducted at Welkite town from 1 February to 30 March 2021. Welkite town is the administrative center of the Gurage zone of the Southern Nations, Nationalities, and Peoples’ Region (SNNPR). The town is located at a latitude and longitude of 8o17′N37o47′E and an elevation between 1910 and 1935 m above sea level (Figure 1). It is located 155 km west of capital city of the county, Addis Ababa. The town has three sub-cities and six kebeles (the smallest administrative unit) and it is one of the most densely populated towns in Ethiopia with an average population density of 283 people/km^2^.

### Study Design and Population

A community-based cross-sectional study design was employed. The source population was all the under-five year children living in Welkite town, and the study population was under-five children living in the selected kebeles of Welkite town.

### Sample Size Determination

Sample size was estimated for a 2 week recall period based on a previous report of 21.3% among under-five children in Halaba special district, in southern Ethiopia [22]. With a 95% confidence level, the margin of error of 5%, and design effect of 1.5 and 10% non-response. This resulted in the final sample size of 426.

### Sampling Technique and Sampling Procedures

**FIGURE 2:**
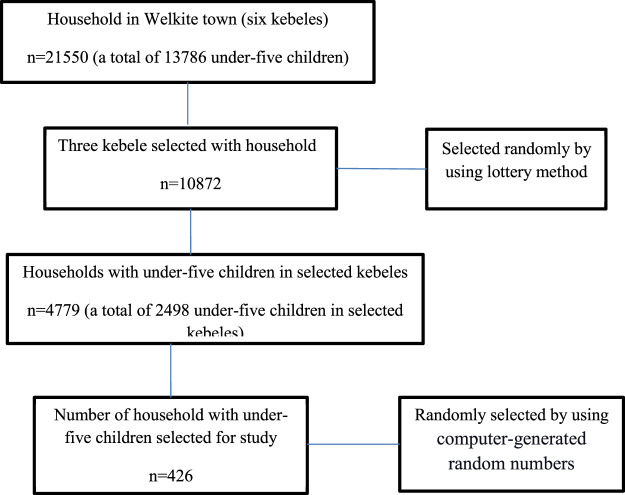
Flow diagram showing recruitment processes of under-five year age children in Welkite town, Gurage zone, southern Ethiopia, 2021.

Multi-stage sampling was used to obtain a representative sample of the study participants. First, three Kebeles (the smallest administrative unit) were selected from the total six kebeles in the town using a random sampling technique. Household lists having under-five children were taken from health extension workers’ registration books in each kebele. Codes were given to each household, and computer-generated random numbers were used to select the households. A lottery method was employed to select one child in case more than one child younger than 5 years old was found in the household (Figure 2).

### Data Collection Methods and Instruments

Socio-demographic, behavioral, environmental, and other related data were collected from mothers/primary caretakers using a structured questionnaire and observation checklists. The questionnaire was adapted from similar studies and customized accordingly. One trained data collector was recruited in each selected kebele and the principal investigator monitored the overall data collection process. Data collectors have been trained about interview methods, consent, and ethical aspects required to be executed during data collection.

### Data Analysis

Data was collected by Open Data Kit (ODK) version 1.27.3 (https://apkpure.com/odk-collect/org.odk.collect.android/download/), and it was exported to SPSS version 25 (IBM Corp., Armonk, NY: IBM Corp.) for analysis. Depending on the distribution of the continuous variables, mean (standard deviation), or median (interquartile ranges) was used to describe the variables, and categorical variables were reported as a number and percentage. The Shapiro-Wilk test was used to evaluate the normality of the quantitative data. Logistic regression was used to identify factors associated with diarrhea status after checking all the preliminary assumptions of the model. Variables with a *p*-value < 0.20 in bivariate analysis were selected as candidate variables to be included in a multivariable analysis in order to control for confounders. Variables with a *p*-value ≤ 0.05 in multivariable analysis were reported as being statistically significant, and AOR with 95% CI was reported as measures of the strength of the associations. The explanatory variables were tested for multi-collinearity using the Variance Inflation Factor (VIF) and the tolerance tests.

### Operational Definitions

Diarrhea is the passage of three or more abnormally loose, watery, or liquid stools over 24 h periods

### Immunization Status

Completely immunized children were those who received all childhood vaccines according to Ethiopia national immunization program for (polio, rotavirus, tuberculous meningitis, and military tuberculosis, pneumonia, diphtheria, pertussis, tetanus, Hemophilus influenza type b, Hepatitis B, and measles).

Partially immunized children were those who received one or more doses of the primary doses of the National Immunization Schedule but not completed all of them.

Safe child stool disposal practice: a child uses a toilet facility or the child’s feces are put into a toilet (regardless of the type of toilet) or buried.

Overcrowding is a condition in which one bedroom is shared by three or more people.

Disposal of household rubbish: Proper solid waste disposal entails burying or storing waste in a container and disposing of it at a designated location. Household trash is improperly disposed of by being placed in open spaces and in roadside ditches along with other garbage.

Environmental sanitation: Environmental sanitation includes human excreta control, managing solid waste and wastewater, and pest and vector control.

## Results

### Socio-Economic and Demographic Characteristics

A total of 426 under-five-year-old children participated in the study with 100% response. Respondents were children’s mothers/primary caregivers and 363 (85.2%) of the respondents were biological parents. The majority 263 (61.8%) of the respondents had primary or secondary levels of education. Of the respondents, 227 (53.3%) were housewives and 101 (23.7%) were government employees. The majority 257 (60.3%) of the study respondents had an independent house with a roof constructed from a corrugated iron sheet. Of the total households included in the study, 352 (82.6%) and 143 (33.6%) had dwellings made of wood and mud wall and mud floor respectively. One hundred ninety-two (45.1%) respondents reported that >4 persons live per household. Households included in this study had 4.57 ± 1.50 SD average family size and each household on average had 2.59 ± 1.21 SD rooms.

The median age of children was 30.5 months with an Interquartile range (IQR) of (19.75–42.25). Most 313 (73.5%) of participants were in the age group of 24–59 months. There were slightly more 217 (50.9%) males than females.

About two-thirds, 266 (62.4%) of the study participants were not breastfeeding at the time of the study but on average they have fed for 21.38± (6.73 SD) months. Most, 331 (77.7%) of participants were fully vaccinated (Table 1).

**TABLE 1:** Socio-demographic characteristics of respondents and study participants in Welkite town, Gurage zone, Southern Ethiopia, 2021.

Characteristics	Response category	Number (%)	95% CI
Relation of the respondent to the child			
	Mothers/father	353 (82.9)	79.3, 86.4
	Sister/brother	49 (11.5)	8.7, 14.3
	Aunt/uncle	24 (5.6)	3.5, 7.7
Sex of children			
	Male	217 (50.9)	46.0, 55.6
	Female	209 (49.1)	44.4, 54.0
Age of children (in month)			
	<6	15 (3.5)	1.9, 5.4
	6–11	17 (4.0)	2.1, 6.1
	12–23	81 (19.0)	15.3, 22.8
	24–59	313 (73.5)	69.5, 77.7
Immunization status of children			
	Fully vaccinated	331 (77.7)	73.9, 81.7
	Partially vaccinated	95 (22.3)	18.3, 26.1
Education status of care givers			
	No formal education	66 (15.5)	12.2, 19.0
	Elementary (1–8)	151 (35.4)	31.2, 39.9
	Secondary (9–12)	112 (26.3)	22.1, 30.3
	Above secondary	97 (22.8)	18.8, 26.8
Occupation of respondents			
	Government employee	101 (23.7)	19.5, 27.9
	Merchant	48 (11.3)	8.2, 14.3
	Student	50 (11.7)	8.7, 14.8
	Housewife	227 (53.3)	48.4, 58.4
Monthly income			
	<1000 ET birr	9 (2.1)	0.9, 3.5
	1,000–3,000 ET birr	173 (40.6)	35.9, 45.3
	>3000ET birr	244 (57.3)	52.4, 62.2

### Behavioral and Environmental Characteristics

Two hundred ninety-two (68.5%) of the respondents had frequent handwashing habits. Of the total respondents, 288 (67.6%), 185 (43.4%), and 255 (59.9%) wash their hands after the toilet, after cleaning, and before feeding the child respectively. Household members of 33 (7.7%), 139 (32.6%), and 190 (44.6%) had the habit of consuming raw milk, raw vegetable, and overnight stored foods after its preparation, respectively. From the observation of household environment, it was found that 206 (48.4%) of water storage area and 88 (20.7%) of water-storing materials were not clean and not covered, respectively. With regards to the food preparation area, 251 (58.9%) households prepare food in the unclean area and there were flies in 89 (20.9%) of households’ food preparation area. The majority 288 (67.6%) of households had improved latrine and almost half 217 (50.9%) of them had private latrine used only by a given household. About three-fourth, 318 (74.6%) of households dispose of their child’s stool in the toilet. Faeces were observed outside the toilet in 71 (16.7%) of the households and there were flies in 145 (34.1%) households. Almost all households 385 (90.8%) had no handwashing facility. There were human and animal faces lying around the house in 10 (2.3%) and 24 (5.6%) of households respectively. Children playing with animal feces lying around the house were observed in 23 (5.3%) of the households (Table 2).

**TABLE 2:** Behavioural and environmental characteristics of respondents in Welkite town, Gurage zone, southern Ethiopia, 2021.

Characteristics	Response category	Number (%)	95% CI
Source of drinking water			
	Tap water	387 (90.8)	88.0, 93.2
	Well/spring	15 (3.5)	1.9, 5.4
	Bottled water	21 (4.9)	3.1, 7.0
	Other	3 (0.7)	0, 1.6
Usual drinking water storing materials			
	Plastic jerrycan with cover	394 (92.5)	89.7, 95.1
	Plastic jerrycan without cover	2 (0.5)	0.0, 1.2
	Other	30 (7.0)	4.7, 9.6
How frequently do you clean your water fetching material?			
	Once per day	77 (18.1)	14.6, 21.8
	Once per week	274 (64.3)	59.9, 68.5
	Once per month	25 (5.9)	3.8, 8.2
	When I see it dirty	50 (11.7)	8.7, 14.8
How frequently do you clean your water storing material?			
	Once per day	38 (8.9)	6.1, 11.7
	Once per week	262 (61.5)	56.6, 65.7
	Once per month	72 (16.9)	13.4, 20.4
	When I see it dirty	54 (12.7)	9.9, 16.0
Kind of toilet facility			
	No facilities or bush or field	3 (0.7)	0.0, 1.6
	Flush toilet	29 (6.8)	4.7, 9.2
	Ventilated improved pit (VIP) latrine	8 (1.9)	0.7, 3.3
	Pit latrine with slab	251 (58.9)	54.2, 63.8
	Open pit latrine	135 (31.7)	27.5, 36.1
How do you dispose the stools of your young children?			
	Child use toilet/latrine	66 (15.5)	12.2, 18.8
	Put in toilet	319 (74.9)	70.7, 78.9
	Put in drain or ditch	16 (3.8)	2.1, 5.6
	Other	25 (5.9)	3.8, 8.2
How do you usually dispose of household rubbish?			
	Rubbish pit	25 (5.9)	3.8, 8.4
	Put in garden	24 (5.6)	3.5, 8.0
	Put in bush	30 (7.0)	4.7, 9.4
	Open burning	189 (44.4)	39.4, 48.6
	Other	158 (37.1)	32.6, 41.8

Respondents from 109 (25.6%) households reported the presence of domestic animals in their house and most 82 (75.2%) reported that animals stay outside the dwelling at night. Of the reported animal type present in the household, 42 (38.5%) and 34 (31.2%) were cats and chickens, respectively. Children had close contact with animals in about one-third 37 (33.9%) of households where the presence of animals was reported.

### Prevalence of Two Weeks Diarrhea and Its Associated Factors

The 2-week prevalence of diarrhea among under-five children was 20.7% (88/426) (95% CI: 17.1%, 24.6%). In bivariate analysis, child's immunization status, mother's/caregiver's educational status, mother's/caregiver's occupation, number of people sharing a sleeping room, breastfeeding, child's stool disposing practice, eating prepared food after overnight storage, cleanness of water storage area, water storage material being covered, cleanness of food preparation area, presence of flies in food preparation area and detection of feces outside a toilet were identified as candidate variables for the multivariable analysis. In the multivariable analysis the odds of having diarrhea among under-five years age children during the last 2 weeks was about five times higher among children whose mothers/caregivers are merchants as compared to children whose mothers/caregivers are housewives [AOR = 5.34; 95% CI: 2.06, 13.82]. Likewise, the odds of a child having diarrhea is 2.67 times higher among partially vaccinated children compared to fully vaccinated counterparts [AOR = 2.67; 95% CI: 1.23, 5.83]. Furthermore, the odds of a child having diarrhea is about two times higher in children living in a household where flies are found in a food preparation area than their counterpart [AOR: 2.24; 95% CI: 1.05, 4.78]. Additionally, the odds of diarrhea occurrence was about five times higher among children whose mothers/caregivers dispose of their stool into garbage than those who put child stools in the toilet [AOR = 5.05; 95%CI: 1.09–23.32]. Children living in households where water was stored without cover were 2.35 times more likely to develop diarrhea than their counterparts ([AOR = 2.35 (1.18, 4.66)]. The difference in the prevalence of diarrhea across categories of potential risk factors as compared to respective reference categories and findings from multi-variable logistic regression analysis are summarized in Table 3.

**TABLE 3:** Risk difference in different categories and Multivariable Logistic Regression analysis results of factors associated with diarrhea among under-five year age children in Welkite town, Gurage zone, southern Ethiopia, 2021.

Characteristics	Number (%) without diarrhea	Number (%) with diarrhea	Risk difference in different categories (95%CI)	AOR (95%CI)	*p*-value
Education status					
No formal education	43 (65.2)	23 (34.8)	0.27 (0.14, 0.39)	0.55 (0.128, 2.368)	0.422
Primary (1–8)	116 (76.8)	35 (23.2)	0.15 (0.06, 0.24)	0.55 (0.163, 1.883)	0.344
Secondary (9–12)	90 (80.4)	22 (19.6)	0.11 (0.02, 0.21)	0.52 (0.152, 1.789)	0.300
Above secondary	89 (91.8)	8 (8.2)	1	**1**	
Respondents Occupation					
Student	37 (74.0)	13 (26.0)	0.04 (−0.09, 0.17)	2.47 (0.949, 6.443)	0.064
Government employee	93 (92.1)	8 (7.9)	−0.14 (−0.22, −0.06)	0.58 (0.182, 1.850)	0.357
Merchant	32 (66.7)	16 (33.3)	0.11 (−0.03, 0.25)	5.34 (2.063,13.817)	0.001*
Housewife	176 (77.5)	51 (22.5)	1	1	
People sleeping in one room		2.92 ± 0.917		1.28 (0.919, 1.782)	0.144
Breast feeding					
No	220 (82.7)	46 (17.3)	−0.09 (−0.173, −0.008)	0.74 (0.312, 1.749)	0.491
Yes	118 (73.8)	42 (26.2)	1	1	
Immunization status					
Partially vaccinated	65 (68.4)	30 (31.6)	0.145 (0.043, 0.246)	2.67 (1.225, 5.826)	0.013*
Fully vaccinated	273 (82.5)	58 (17.5)	1	1	
Child stool disposal					
Put in toilet	254 (79.6)	65 (20.4)	1	1	
Child use toilet/latrine	56 (84.8)	10 (15.2)	−0.05 (−0.15, 0.05)	0.667 (0.239, 1.860)	0.439
Thrown into garbage	7 (43.8)	9 (56.2)	0.36 (0.11, 0.61)	5.05 (1.094, 23.324)	0.038*
Others	21 (84.0)	4 (16.0)	−0.04 (−0.20, 0.11)	0.251 (0.040, 1.570)	0.139
Eating prepared food after overnight storage					
No	199 (84.3)	37 (15.7)	1	0.69 (0.369, 1.278)	0.236
Yes	139 (73.2)	51 (26.8)	−0.16 (−0.10, 0.07)	1	
Cleanness of water storage area					
No	139 (67.5)	67 (32.5)	0.23 (0.16, 0.31)	1	
Yes	199 (90.5)	21 (9.5)	1	2.06 (0.854, 4.956)	0.108
Water storage material being covered					
No	49 (55.7)	39 (44.3)	0.30 (0.18, 0.41)	2.35 (1.183, 4.662)	0.015*
Yes	289 (85.5)	49 (14.5)	1	1	
Cleanness of food preparation area					
No	174 (69.3)	77 (30.7)	0.24 (0.18, 0.31)	1.76 (0.650, 4.777)	0.266
Yes	164 (93.7)	11 (6.3)	1	1	
Flies in food preparation area					
No	278 (82.5)	59 (17.5)	1	1	
Yes	60 (67.4)	29 (32.6)	0.15 (0.05, 0.26)	2.24 (1.054, 4.779)	0.036*
Feces outside toilet					
No	296 (83.9)	57 (16.1)	1	1	
Yes	42 (57.5)	31 (42.5)	0.26 (0.14, 0.38)	2.13 (0.970, 4.691)	0.059
Flies around a toilet					
No	246 (87.5)	35 (12.5)	1	1	
Yes	92 (63.4)	53 (36.6)	0.24 (0.15, 0.33)	1.59 (0.806, 3.159)	0.180

1 = reference category, * statistically associated *p*-value <0.05.

## Discussion

This study assessed the burden and risk factors of diarrhea among under-five children in Welkite town. In this study, 20.7% of under-five children were reported to have diarrhea in the 2 weeks. This result is comparable with the study in Dakahlia, Egypt (23.6%) [23] and Malawi (20.7%) [24]. It is also comparable with the finding of studies conducted in different localities in Ethiopia such as Jamma district (23.1%) [25], Eastern Ethiopia (22.5%) [26], and Jabithenan district (21.5%) [27].

The magnitude of diarrhea in this study is higher than the Ethiopian national prevalence of diarrheal disease (12%) in under-five children reported by EDHS 2016 [28]. It is also higher than a study conducted in Dale district Sidama zone (13.6%) [20], Debre Brehan (16.4) [29], Kamashi district (14.5%) [30], and India (9.0%) [31]. However, it was lower than the finding from Arbaminch (30.5%) [32], Guji zone, Oromia region (36.5%) [33], Tigray region of Northern Ethiopia (27.2%) [34], Kashmir, India (25.2%) [35], Mbour, Senegal (26.0%) [36] and Northern Uganda (29.1%) [37]. The difference might be attributed to the variation in the socio-demographic, behavioral, and environmental factors of study households.

Most of the time paper currencies are contaminated with pathogenic microorganisms and they could be one of the most potential vehicles to transmit pathogens amongst people [38]. Proper handwashing is one of the most effective ways of preventing the spread of diarrheal diseases. In this study, mothers’/caregivers’ occupation is identified as an independent predictor of diarrhea. The study revealed that the likelihood of having diarrhea is about five times higher among under-five children if the mothers’/caregivers’ occupation is a merchant as compared to a housewife. This might be related to the fact that merchants have frequent contact with paper currencies and people coming to buy and sell their products. They also have a high probability of hand contamination during handling items at their workplace. However, hand washing practice between money transactions was not reported by any respondent in this study. This may play a significant role in the transmission of diarrheagenic pathogens to their children. In addition, merchant mothers may not have enough time to take care of their children during the daytime, which may lead their children to poor hygiene and nutritional status.

Incomplete immunization status was also another risk factor identified as a predictor of diarrhea prevalence in children. In this study, the odds of having diarrhea is about three times higher among partially vaccinated children compared to fully vaccinated ones. This finding is in line with the study done in Ethiopia [33, 39] and West Bengal [40]. This may be because immunization can reduce mortality and morbidity from common childhood diseases including diarrhea by increasing their capability to combat disease. Among childhood vaccines, the rotavirus vaccine can directly prevent infections that cause diarrhea and measles vaccines can prevent infections that can lead to diarrhea as a complication of an illness [5].

Hygienic food preparation is recommended as a preventive measure for the control of diarrhea. This study indicated that the presence of flies in a food preparation area was positively associated with the occurrence of diarrhea. Children living in households where flies are found in food preparation areas were about two times more likely to develop diarrhea compared to the other group. This might be explained by the fact that the presence of houseflies in a food preparation area increases the risk of food contamination through direct contact with food by walking on the food or through their droppings. They are known to carry and transmit diarrhea-causing agents by mechanical transfer from the exoskeleton, regurgitation, and fecal deposit. Some enteropathogens can multiply in the gut of houseflies and can be excreted for more than weeks [41, 42]. This result is consistent with the study done in Ghana [43].

Safe disposal of feces is important to reduce the risk of contact between causative agents of diarrhea and the host. Unsafe disposal of child feces has been associated with an increased risk of diarrheal diseases. Disposal of child feces in the domestic environment can provide breeding sites for flies, which are known vehicles of diarrheal pathogens [44]. Inappropriate child stool disposing behavior was significantly associated with diarrhea occurrence in this study with more children having diarrhea where their mothers/caregivers throw their stool into the garbage. According to this study, the likelihood of developing diarrhea in children is 5.05 times higher if households are disposing child feces into the garbage as compared to households using latrines for disposal. The finding is consistent with a case-control study conducted in Ethiopia [45]. A study in Burkina Faso also reported that regular disposal of child stool in the latrines had an approximate 40% reduction in the diarrhea rate [46].

Diarrhea can be reduced significantly if water quality can be ensured up to the point of consumption. The risk of water contamination is high if the storage materials are not covered and it poses a greater risk of diarrhea. A significant association between diarrhea in under-five children and the habit of not covering drinking water storage material was revealed in this study. Children living in households where water was stored without cover were 2.35 times more likely to acquire diarrhea than their counterparts. This is consistent with the WHO report that showed the effective and consistent application of household water treatment and safe storage can reduce diarrheal diseases by between 28% and 45%, depending on the type of water supply [47].

One of the limitations of the current study is the inability to assess the nutritional status of children and the inability to establish a causal link between diarrhea and independent predictors due to the cross-sectional nature of the study design. In addition, the pattern of diarrhea cases in different seasons was not studied. Furthermore, because the respondents self-reported the occurrence of diarrhea and several behavioral habits like hand washing habits, they may have introduced bias into the estimates.

In conclusion, the prevalence of childhood diarrhea is high in the study area. Mothers/caregivers’ being merchants, children’s partial immunization status, throwing away a child’s stool into the garbage, not covering water storage materials, and the presence of flies in the food preparation area were all independent predictors of diarrhea. Therefore, targeting identified risk factors with special attention is useful to reduce the occurrence of diarrhea in under-five children. Besides, further studies using qualitative methods are needed to explore local and cultural beliefs and practices of mothers/caregivers. It may provide a better understanding of the nature of epidemiology of under-five diarrhea.

## Data Availability

Data will be available from the corresponding author upon a reasonable request.

## Abbreviations

AOR, Adjusted Odds Ratio; ODK, Open Data Kit; WHO, World Health Organization.
